# Realizing the potential of full-length transcriptome sequencing

**DOI:** 10.1098/rstb.2019.0097

**Published:** 2019-10-07

**Authors:** Ashley Byrne, Charles Cole, Roger Volden, Christopher Vollmers

**Affiliations:** 1 Department of Molecular, Cellular, and Developmental Biology, University of California Santa Cruz, Santa Cruz, CA 95064, USA; 2 Department of Biomolecular Engineering, University of California Santa Cruz, Santa Cruz, CA 95064, USA

**Keywords:** transcriptome analysis, long-read sequencing, Oxford Nanopore Technologies, Pacific Biosciences

## Abstract

Long-read sequencing holds great potential for transcriptome analysis because it offers researchers an affordable method to annotate the transcriptomes of non-model organisms. This, in turn, will greatly benefit future work on less-researched organisms like unicellular eukaryotes that cannot rely on large consortia to generate these transcriptome annotations. However, to realize this potential, several remaining molecular and computational challenges will have to be overcome. In this review, we have outlined the limitations of short-read sequencing technology and how long-read sequencing technology overcomes these limitations. We have also highlighted the unique challenges still present for long-read sequencing technology and provided some suggestions on how to overcome these challenges going forward.

This article is part of a discussion meeting issue ‘Single cell ecology’.

## 1. Introduction

The rapid progress and application of sequencing technology after the completion of the Human Genome Project has led to a vastly expanded knowledge of the genome sequences present in the eukaryotic tree of life. However, owing to the cost and technological limitations, truly high-quality genome references have been limited to a core of organisms of large scientific or economic interest. Further, our knowledge of which parts of genomes constitute genes and which transcript isoforms these genes produce, i.e. high-quality transcriptome annotation, is even more scarce [1]. As a result, much of the diversity of transcriptomes in less-researched organisms like unicellular eukaryotes remains unexplored.

Transcriptome annotations are of great value to researchers because they are required for us to understand how genome sequences and changes to these sequences are interpreted by the cellular machinery. They are also required for many functional analyses. Without accurate transcriptome annotations, we cannot, for example, perform RNA-seq experiments to investigate differential expression of genes or predict which proteins are present in a tissue or organism.

Now, sequencing technology is reaching a point where it might soon be feasible to affordably generate high-quality genome references and transcriptome annotations of a much wider range of organisms than previously possible—including unicellular eukaryotes. However, while genomes will soon be relatively reliably assembled into chromosome-scale scaffolds, technological limitations cause transcriptome annotation approaches to lack behind in their ability to identify genes and their isoforms expressed from these chromosomes.

Genome assembly is now entering a golden age where high-quality ‘centromere-to-telomere’ genome sequences can be assembled through a mix of several technologies, including short-read sequencing, linked short-read sequencing (e.g. Hi-C), long-read sequencing and optical mapping [2–4]. These powerful and relatively affordable approaches are going to be of outsize benefit for non-model organisms from unicellular eukaryotes to polar bears that in the past did not receive the attention and large sums of money required to generate a high-quality genome reference the hard way—chromosome maps, Sanger sequencing of bacterial artificial chromosome libraries, etc.

Most of these advances in genome assembly are not transferable to transcriptome annotation. Currently, short- and long-read sequencing protocols are used for transcriptome annotation but have underlying limitations that make reaching a ‘reference-level’ transcriptome annotation both highly labour intensive and often simply impossible.

Here, we discuss the potential and limitations of long-read based full-length transcriptome sequencing for transcriptome annotation and lay out a path towards realizing this potential.

## 2. What are the limitations of short-read sequencing technology?

The analysis of what RNA transcripts (annotation) is present in a sample and at what level (quantification) has relied on a mix of technologies over the last three decades. Early efforts to annotate and quantify complex eukaryotic transcriptomes were highly labour intensive. During the early 1990s, efforts to evaluate RNA sequences on a large scale relied heavily on expressed sequence tags (ESTs), whereby complementary DNA (cDNA) molecules were individually cloned, screened and Sanger-sequenced to determine full-length mRNA sequences and observe semi-quantitative changes in gene expression [5]. The Sanger-sequencing based serial analysis of gene expression (SAGE) method improved quantification and reduced cost by concatenating smaller 15–20 bp fragments of many cDNA molecules together for sequencing [6]. However, because of the short length of analysed fragments SAGE was inherently less useful for annotation. Hybridization-based microarray approaches completely eschewed annotation but simplified the quantification of already annotated genes [7].

The introduction of massively parallel sequencing in the mid-to-late 2000s completely changed transcriptome annotation and quantification. When massively parallel sequencing—best represented by the now dominant Illumina technology—became available to research laboratories it could generate millions of sequencing reads at a length of approximately 30 nucleotides (nt). Although initially intended for the sequencing of genomic DNA, researchers quickly found ways to leverage the power of these sequencers for transcriptome analysis in the form of the RNA-seq assay. RNA-seq sequences short cDNA fragments at extremely high throughput and quickly displaced microarray-based transcriptome analysis for a number of reasons including cost considerations as well as the ability to detect previously unknown transcripts and quantify the use of individual splice sites. In the last decade, Illumina sequencers have steadily and massively improved, although these improvements have come with compromises in experimental design. Most prominently newer Illumina sequencers require additional precautions to avoid sample cross-contamination during the sequencing reaction [8].

Current Illumina sequencers like the NovaSeq can generate billions of sequencing reads at a length of 150 nt allowing the multiplexed analysis of hundreds to thousands of samples in a single run (table 1). At this read length and output, RNA-seq reads are not only useful for transcriptome quantification but also for annotation. Consequently, efforts like GENCODE and RefSeq heavily rely on this data type for their respective annotation approaches [11,12]. Paired with literally hundreds of sample preparation techniques and analysis pipelines, transcriptome analysis by short-read RNA-seq [13] is now a core component of research in nearly all fields of biology.

**Table 1.** Sequencing technology characteristics. (Read number per dollar is hard to establish considering different pricing structures and instrument costs. Here, we assume a laboratory would use sequencing cores for Illumina and PacBio sequencing while performing ONT MinION sequencing themselves.)

technology	read number/$1 k	read accuracy (%)	consensus accuracy
Illumina NextSeq	∼2 × 10^8^	99.9	N/A
Pacific Biosciences (PacBio) sequel	∼4 × 10^5^	89	>99%
Oxford Nanopore Technologies (ONT) MinION	∼5 × 10^6^	88	>97.5%^a^

^a^Consensus accuracy using our R2C2 approach as published [9,10].

So, while it is clear that RNA-seq has revolutionized transcriptome annotation and quantification, it is also becoming increasingly clear that it is ultimately a stop-gap solution of limited power born out the limitations of short-read sequencing. These limitations prevent RNA-seq from annotating and quantifying transcriptomes on the level of RNA transcript isoforms, i.e. transcript variants expressed by the same gene using combinations of alternative splice sites, transcription start sites (TSSs) and transcription termination or polyA sites. Thus, to fully understand the fundamentals of gene expression, isoform information will be required.

### (a) Limitations in transcriptome assembly algorithms

**Figure 1.**
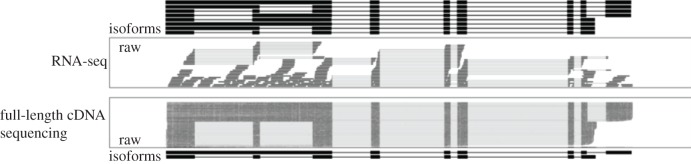
Fundamental difference between short- and long-read sequencing of transcripts. Short RNA-seq reads only capture small fragments of transcripts. RNA-seq data, therefore, lacks unambiguous isoform data leading to the inference of many erroneous isoforms. Long-read full-length cDNA data captures transcripts end-to-end making isoform inference unambiguous.

Despite its dominant position in transcriptome analysis, short-read RNA-seq has so far failed at capturing the true complexity of eukaryotic transcriptomes. While RNA-seq can interrogate individual transcript features like splice sites, TSSs and polyA sites, it fails at determining how these individual features are combined into transcript isoforms. This is owing to the fact that the read length of short-read sequencers is too short to capture entire transcripts from end-to-end (figure 1). Incomplete fragments of transcripts, therefore, have to be computationally assembled into full-length isoforms. This is done using powerful algorithms performing de novo (e.g. Trinity, rnaSPAdes [14,15]) or genome-guided transcriptome assemblies (e.g. Cufflinks, StringTie [16,17]). All of these assemblers ultimately fail at discerning complex transcript isoforms expressed by the same gene because of limitations of the underlying data. First, RNA-seq reads often do not cover the ends of transcripts leaving TSS and polyA sites unresolved [18]. Second, alternative transcript features are too far apart to be resolved by RNA-seq raw data, i.e. if a transcript has two alternative splice sites 1000 bp apart, no individual RNA-seq read will ever connect those two events. Computational methods that take this into account have been developed, however, they still fail at deconvoluting complex isoform mixtures [19].

### (b) Limitations in short-read sequencing technology

To date, no RNA-seq protocol has succeeded in providing data capable of overcoming this assembly challenge and recovering full-length isoforms in a high-throughput manner. Although short-read sequencing technology has increased its sequencing length capability to approximately 300 nt from the original approximately 30 nt, it still cannot sequence the vast majority of transcripts from end-to-end. To get around the read length limitation, creative specialized protocols have been developed. The most successful methods include synthetic long read (SLR) and spISO-seq, which operate on the principle of splitting one sample into hundreds or thousands of separate reactions using either 384-well plates or microdroplets [20,21]. This separation allows the generation of individual sequencing libraries that ideally only contain one transcript isoform for any specific gene. These libraries can then be sequenced and analysed separately which massively simplifies computational assembly and reduces misassemblies. However, while improving on general RNA-seq methods, neither method succeeds at efficiently generating transcript isoforms. SLR generates a low number of transcripts most of which are incomplete at the 3′ end, while spISO-seq generates sparse ‘read-clouds’ that can connect individual splice sites but fail at consistently capturing 5′ and 3′ ends of transcripts. Additionally, both SLR and spISO-seq approaches have complex library preparation workflows that cannot be multiplexed and require specialized instrumentation which has prevented them from being widely adopted.

While it is not inconceivable that a future short-read based protocol will ultimately succeed at isoform-level analysis, this task currently appears to be well beyond the capabilities of short-read sequencing.

## 3. How can the potential of long-read transcriptome sequencing be realized?

We believe that long-read sequencing is on the verge of disrupting transcriptome analysis similarly to how short-read sequencing did a decade ago. In contrast with short-read sequencing, long-read sequencing technology as provided by Pacific Biosciences (PacBio) and Oxford Nanopore Technologies (ONT) has the potential to identify and quantify isoforms simply by sequencing cDNA or mRNA molecules end-to-end from 3′ polyA tail to 5′ cap.

Just like short-read sequencing, long-read technology was initially intended for genomic DNA sequencing, but it was only a matter of time until cDNA copies of RNA transcript molecules were sequenced on PacBio and ONT sequencers.

Initial studies used long reads for the targeted analysis of specific highly complex transcripts [22] or to add small amounts of long-read data to short-read RNA-seq data [23,24]. Increasing read throughput has allowed the analysis of whole transcriptomes of diverse organisms with long-read data alone [9,25–27] and in addition to the analysis of cDNA, ONT sequencers now offer the ability to sequence RNA directly [28,29]. Finally, long-read technology has been used to analyse the transcriptomes of single cells [30–32].

These papers clearly highlight the potential of long-read sequencing to identify new isoforms and isoform features like new splice sites, TSSs and polyA sites, which is essential to unambiguously annotate and quantify transcriptomes. These papers also lay out a path for the future: in the short-term, long-read technology will be a boon for the transcriptome annotation of non-model organisms. With a moderate investment generating long-read transcriptome data for a variety of tissues and organs present in a non-model organism, transcriptome annotations will get close to the comprehensiveness and quality of highly curated mouse and human transcriptomes. In the long-term, we believe long-read technology has the potential to entirely replace short-read RNA-seq for transcriptome analysis.

However, to realize this potential, long-read transcriptome analysis still has to overcome several challenges that are currently limiting its progress.

## 4. What are the challenges of long-read sequencing?

Although the above examples have highlighted the potential for long-read technology, there still remains significant challenges which affect both PacBio and ONT to varying degrees: (a) RNA integrity, (b) length bias, (c) read throughput, (d) read accuracy, and (e) data analysis.

In order for long-read sequencing to be a main driver in pushing the transcriptome field forward these challenges will have to be overcome.

### (a) RNA integrity

All current long-read transcriptome sequencing approaches suffer from experimental artefacts caused by degraded RNA molecules.

While ONT and PacBio sequencers make it possible to sequence entire transcripts end-to-end, this only matters if the vast majority of sequenced transcript molecules are fully intact. The integrity of RNA going into long-read sequencing experiments is therefore of the highest importance. However, it is not yet clear what represents the best extraction and processing method for RNA.

Single-cell studies circumvent this issue by performing reverse transcription (RT) directly on cell lysates resulting in high-quality results [30,32], but this is not possible for bulk samples comprising tissues or many cells because highly concentrated cell lysates inhibit RT reactions. Current efforts to dissociate, lyse and extract RNA from bulk samples mostly rely on physical disruption and trizol or tri-reagent based protocols. These protocols are either followed by precipitations often resulting in phenol and guanidium contamination which can compromise RNA integrity, or require a column-based clean-up potentially fragmenting long RNA transcripts in a way similar to high molecular weight genomic DNA.

Going forward we will need systematic studies comparing extraction methods for the integrity for very long transcripts (greater than 10 kb) which cannot be measured by the frequently used RNA Integrity Number value which is calculated by evaluating the integrity of the much shorter rRNA transcripts at approximately 2 kb (18S) and approximately 5 kb (28S).

We believe these efforts are likely to succeed. Moving from short-read to long-read sequencing has already led to the genomics community rethinking the way it extracts DNA—from mostly column-based to precipitation-based approaches—leading to the successful sequencing of DNA molecules almost 1 million base-pairs in length [2].

### (b) Length bias

All current long-read transcriptome sequencing approaches are biased towards short transcripts. As a result, the lengths of reads produced and therefore transcripts sequenced by these various approaches do not reflect the transcript lengths as determined by annotation efforts like GENCODE. While the expression of short and long transcripts surely varies for each sample and each sample will only include a fraction of all transcripts in the GENCODE annotation, the fact remains that current long-read approaches appear to have a hard time capturing long transcripts.

This bias is rooted in the way samples are prepared for sequencing as well as the sequencing technology itself. To prepare full-length eukaryotic mRNA molecules for sequencing, protocols for PacBio and ONT sequencers today rely on some version of RT using oligo-dT priming most often paired with template switching as featured in the Smart-seq2 protocol [18]. This RT step generates cDNA with known 3′ and 5′ ends that can be polymerase chain reaction (PCR) amplified. PCR amplification is required to generate enough cDNA for sequencing library preparation—several micrograms for either technology. However, if cDNA is PCR amplified, short transcripts are more likely to be successfully amplified, thereby generating a pool of cDNA skewed towards full-length short transcripts (less than 2 kb) and shorter amplification artefacts of long transcripts.

**Figure 2.**
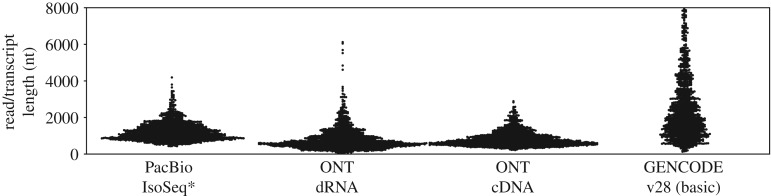
Long-read transcriptome sequencing approaches do not cover long transcripts. Swarmplots of length distributions of 1000 randomly sampled PacBio [9], ONT dRNA and cDNA [28] reads covering the GM12878 (human lymphoblast cell line) transcriptome. These distributions are not representative of the length distribution of the human transcriptome as annotated by GENCODE. *While we show the most recent dataset on GM12878 we could find for PacBio technology it is several years old and might not be fully representative of current platform performance.

While ONT sequencers can now sequence RNA directly, recent studies have shown that this does not overcome RNA degradation or length-bias issues. In fact, incomplete transcript sequences represent the majority of the produced data and this issue increases with increased transcript length making direct RNA sequencing currently challenging for transcripts over 2 kb in length (figure 2) [28].

In addition to length biases of sample preparation, both PacBio and ONT sequencers themselves have a bias towards shorter molecules.

To systematically test the bias of different RNA extraction, sample preparation and sequencing methods, new approaches will be needed. Unfortunately, current synthetic RNA spike-in mixtures like External RNA Controls Consortium and spike-in RNA variant control mixes (Lexogen), only contain molecules less than 2.5 kb which is simply not long enough to determine bias against long transcript molecules (figure 2). To truly determine length bias, it would require sequencing of a well-defined eukaryotic human transcriptome, e.g. human cell line GM12878, using an RNA molecule length independent short-read RNA-seq method. While short-read RNA-seq would not be able to systematically resolve isoforms, assemblies of these reads can be used to estimate transcript lengths. Comparing representation of transcripts of different lengths in long-read datasets prepared with different protocols will then help reveal biases of these protocols.

The question then still remains: how do we overcome the inherent limitations of PCR amplification, sequencing library preparation, and the cDNA and direct RNA sequencing process itself. One thing that is certain is that future efforts will have to overcome these limitations or, ironically, the world of long transcripts will remain closed to long-read transcriptomics.

It will be up to the wider genomics community as well as PacBio and ONT to address these limitations. While adding complexity to sample preparations and distorting sample compositions, size selections on the RNA or cDNA level might mitigate length bias in sample preparation. Also, reducing cDNA amounts required for sequencing reactions might eliminate the need for PCR entirely. Additionally, PacBio sequencers have already made big strides reducing the length bias of their library preparation and sequencing reactions in the last few years and it would be surprising if this was not a big focus of ONT as well. Finally, one way to get around the length-bias of sequencing library preparation and sequencing reactions themselves is to dissociate transcript length from the length of the DNA/RNA being sequenced, i.e. making all DNA/RNA going into a sequencing reaction approximately the same length. This could be done by randomly ligating transcripts into large chimeric molecules or generating large DNA concatemers containing many copies of the same transcript [31].

### (c) Read throughput

All sequencing-based transcriptome analysis is ultimately limited by the number of reads available for analysis.

More reads result in better data but so far there has not been a rigorous study to identify the exact numbers of long sequencing reads required to exhaustively analyse a complex eukaryotic transcriptome. Because sequencing-based transcriptome analysis follows the same sampling principle regardless of read length, it stands to reason that these numbers will be similar to those required for short-read RNA-seq assays. Therefore, greater than 30 million reads will be required for a shallow analysis of a transcriptome of a bulk sample capturing the isoforms of genes with medium and high expression [33]. This, however, represents an ideal scenario assuming a single isoform per gene. If we think about treating individual isoforms as individual genes, it follows that significantly deeper sequencing will be needed to identify and quantify them. Indeed, it has been suggested by a deep-sequencing survey of alternative-splicing in human tissue that there are, on average, seven alternative splicing events per multi-exon gene [10]. Therefore, to truly explore the complexity of mammalian transcriptomes, greater than 100 000 000 of reads covering full-length transcripts will be required per tissue or organ.

In contrast with bulk samples, estimating the read depth required for single cell analysis is more straightforward as it is limited by experimental constraints. Most workflows in the rapidly expanding field of single cell transcriptome analysis attach unique molecular identifiers to each cDNA molecule generated for each individual cell, thereby giving us a direct way to determine the number of reads needed to capture all or most of these molecules [34]. 10X Genomics single cell analysis approach, for example, generates less than 20 000 cDNA molecules per cell [35]. To reliably capture greater than 90% of these molecules, sampling statistics dictate the need for approximately 45 000 sequencing reads per cell and consequently 45 000 000 sequencing reads for the analysis of a 1000 cell cDNA pool.

In short, future long-read transcriptome analysis of bulk and single cell samples will require tens to hundreds of millions of reads at a reasonable cost. While ONT sequencers now routinely generate several millions of reads per $1000 of sequencing, PacBio sequencers produce approximately 400 000 reads per $1000 of sequencing (table 1). This means that achieving the sequencing depth required for exhaustive transcriptome analysis is now borderline feasible with ONT sequencers but would deplete all but the largest of research budgets if using PacBio sequencers.

It is going to be highly interesting to see how the newly released ONT PromethION and PacBio Sequel II will change this equation once in researchers' hands as they both represent significant improvements in read throughput over the ONT MinION and PacBio Sequel, respectively.

### (d) Read accuracy

As long as a PacBio or ONT read captures the sequence of a full-length transcript and is accurate enough to be correctly aligned to a single genomic location, it is useful for analysis. There is no line in the metaphorical read-accuracy sand beyond which this transcript sequence becomes useless for analysis, because different downstream applications will require different levels of accuracy to be implemented. It is, however, no surprise that more accurate reads are always preferable over less accurate reads.

Both PacBio and ONT long-read technologies sequence individual DNA (or RNA) molecules and as such are inherently more error-prone than short-read Illumina sequencing which can rely on the combined signal of thousands of copies of DNA molecules to determine the base sequence. Because the raw read length of PacBio sequencers is much longer than an average transcript molecule, circularized cDNA molecules can be read multiple times to generate a more accurate consensus. As a result, PacBio's IsoSeq protocol generates cDNA circular consensus sequences (CCS) that can achieve greater than 99% (Q20) accuracy (table 1) [9,30].

**Figure 3.**
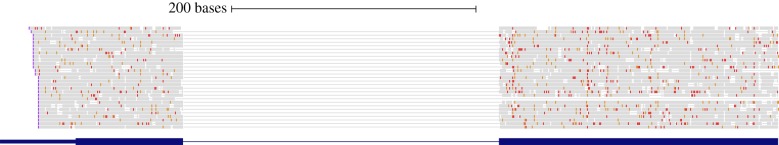
Error-prone reads pose analysis challenge. Representative alignments of ONT cDNA [28] reads. Thirty read alignments (grey) to the first two exons of the CD19 gene (dark blue) are shown. Read alignments contain many insertions (orange), mismatches (red) and deletions (thin line) within exons. These errors complicate the detection of exact transcript sequences and exact positions of splice sites, TSSs and polyA sites.

While ONT raw read length far exceeds transcript length, there currently exists no commercial product to—like PacBio's CCS approach—take advantage of this read length to improve read accuracy through consensus generation. Because of this, cDNA or direct RNA sequencing on ONT (1D) generates sequences of 88% (Q9) accuracy (figure 3).

This low accuracy creates some serious drawbacks regarding downstream analysis including the inability to accurately demultiplex single cell data. This is problematic because working with single cells will be required for the analysis of unicellular organisms that are not culturable in a laboratory environment.

Single cell approaches like 10X Genomics Chromium workflow or the Drop-seq protocol can process many hundreds to thousands of cells in parallel using water-in-oil emulsions to produce highly multiplexed single cell cDNA pools [35,36]. In this process, cell-specific identifiers—short nucleotide sequences—are attached to each cDNA molecule that is reverse transcribed from mRNA. Consequently, assigning a cDNA molecule to the cell it originated from, i.e. demultiplexing, requires accurately determining the sequence of its cell-specific identifier. Without sufficiently accurate sequencing, molecules will therefore be mis-assigned or lost [30].

ONT are working to improve their basecalling accuracy and have announced a commercial consensus approach to be released in 2019 that should address this issue. Until then, the ONT research community including our own laboratory has recognized this issue and developed consensus sequencing approaches [37]. Specifically targeted for cDNA, the R2C2 approach we developed circularizes cDNA and uses rolling circle amplification to generate long concatemeric molecules that can be sequenced and processed into consensus sequences [31]. At $1000 sequencing cost the R2C2 approach can currently produce several million sequencing reads at greater than 97.5% (∼Q16) median accuracy [38].

It is unclear whether ONT consensus approaches will be able to reach the accuracy of PacBio circular consensus reads because, while errors in PacBio sequencing data are not entirely random, they are less systematic than ONT errors [39]. Systematic errors which recur in the same base context, e.g. around homopolymers (stretches of the same base longer than 5 nt) can pose insurmountable challenges for consensus-based error-correction. Error-correcting algorithms like *Nanopolish* [40], *Racon* [41] or *Medaka* [42] are beginning to address this by either making use of the ionic current based raw signal generated by ONT sequencers or by incorporating ONT-specific error models.

While it remains to be determined what accuracy will be sufficient for reliably identifying regular transcript isoforms, increasing the accuracy of individual reads to beyond 99% will not only be required for single cell cDNA demultiplexing but also the analysis of individual transcripts that contain unique sequences not encoded in the genome, i.e. B and T cell receptor transcripts, as well as transcripts that contain base modifications.

### (e) Data analysis

The goal of long-read transcriptome analysis is twofold. First, it aims to identify all transcript isoforms present in a sample, then quantify their expression (ideally in an allele-specific manner).

In contrast with short-read RNA-seq where bioinformaticians have spent the last decade creating a large number of tools for data analysis steps including read alignment, expression quantification and transcriptome assembly, the tools for long-read analysis are still in their infancy. Although long-read technology circumvents many of the bioinformatic assembly challenges of short-read data, error-prone long-read data has created its own new set of challenges. These challenges have necessitated new algorithms for the efficient analysis of longer reads.

Nevertheless, interest among bioinformaticians towards long-read technology is steadily increasing and off-the-shelf tools to analyse long reads are being developed and published. Below is an overview of what the current state of tools is and what we perceive the outstanding challenges in the long-read cDNA field are.

#### (i) Aligning long-read data

**Figure 4.**
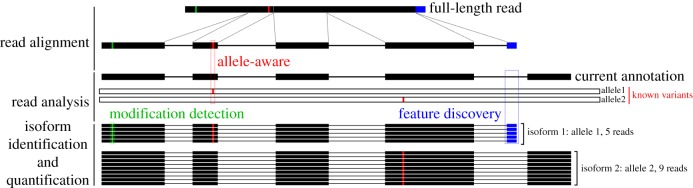
Analysis challenges of long-read full-length sequencing. A simplified schematic shows the steps required to extract information out of long-read sequencing data. Each read has to be aligned, ideally in a allele-aware manner to the genome it originated from. Read alignments then have to be analysed to identify RNA modifications as well as new isoform features that are missing in the current transcriptome annotation. For each allele, reads then have to be grouped into isoforms which allows isoform identification and quantification. For real datasets, all these steps have to take into account the often substantial rates of sequencing errors and incomplete reads in long-read sequencing. These will complicate all steps of the analysis.

Aligning reads to genome sequences is at the core of most transcriptome analysis (figure 4). Luckily there are several good options available for the spliced alignment of noisy long reads. The *GMAP* [43] and *BLAT* [44] aligners, originally developed for the alignment of ESTs perform surprisingly well for aligning noisy long reads. However, just like the PacBio developed *BLASR* [45] aligner, they are simply too slow for the effective analysis of millions of reads. The recently released *minimap2* [46] aligner seems to address the issue of speed while maintaining alignment accuracy and has quickly been adopted among the ONT community. The only trade-off we have observed (however not systematically investigated) is that *minimap2*—potentially owing to the relatively large default seed size of 15 nt—seems to lack sensitivity when aligning reads to very short terminal exons. We hope that future improvements in long-read accuracy will allow alignment algorithms to ‘dial in’ that trade-off between avoiding spurious short alignments and detecting even the shortest of potentially un-annotated terminal exons.

#### (ii) Isoform identification

Transcriptome annotation includes the identification of new gene features as well as how these new features are combined with known features into isoforms (figure 4). This is where long-read transcriptome sequencing holds the largest promise. However, the tools available for the identification isoforms from long read data are still in their infancy.

While PacBio supplies the *IsoSeq3* analysis pipeline for the analysis of their cDNA CCS reads, previous work indicates that this pipeline tends to over-report potential isoforms [47]. There currently exist three pipelines for the analysis of ONT cDNA or direct RNA sequencing data. Both *Pinfish* released by ONT and *FLAIR* released by the Brooks laboratory at University of California Santa Cruz (UCSC) are intended for regular 1D ONT data and deal with the high error-rate in different ways. Of these two pipelines, only *FLAIR* has been used in a publicly available manuscript and deals with inaccurate ONT reads by using short-read Illumina reads to correct splice junctions and identifies and quantifies isoform data; however, it does not use nanopore reads for de novo splice site detection and relies on annotation and short-read data [28].

Specifically designed for the analysis of R2C2 reads, the *Mandalorion* pipeline developed by our laboratory at UCSC takes advantage of the higher accuracy of R2C2 reads to identify and quantify isoforms without the need for Illumina data, while also identifying new gene features and isoforms [31].

One thing to consider when identifying isoforms is how to deal with raw data containing molecular biology artefacts. First and foremost, this includes the amplification of either fragmented RNA or genomic DNA. While, ideally, these artefacts should be minimized during sample preparation, any pipeline should be equipped to recognize potentially incorrect isoforms stemming from them. Tools like *Sqanti* which can detect these types of artefacts can serve as quality control for future isoform identification pipelines [48].

#### (iii) Isoform quantification

Quantifying and performing differential expression analysis of transcript levels on the isoform instead of the gene level brings with it a large set of new challenges.

First, it will be a challenge to decide at which point a known and a newly identified isoform should be treated as the same or equivalent isoforms. Containing different splice sites surely differentiates isoforms, but whether different TSSs that are three nucleotides apart and reside within the 5′ untranslated region differentiate isoforms is not at all clear.

*FLAIR* and *Mandalorion* deal with this by analysing all samples that have to be compared at the same time to create a shared list of isoforms. This creates large computational overheads because adding a single sample to a dataset requires the reanalysis of the entire dataset.

Second, it will be a challenge to systematically differentiate allele-specific isoform expression (figure 4). To differentiate alleles, we will need accurate and phased information of sequence variants differentiating the haplotypes present in a sample, because extracting this information from error-prone long-read transcriptome data is inherently suboptimal. However, if sequence variants are known, tools like *HapCUT2* can be used to assign full-length cDNA to parental alleles [49]. This in principle allows for allele-specific expression analysis.

We are, however, optimistic that approaches that sort aligned reads based on the variants they contain are only a temporary solution. In the future, it is likely that alignment algorithms will be able to take advantage of fully diploid genome sequences during alignment to immediately align reads to the haplotype they originate from. Then, ideally, future tools will identify allele-specific isoforms based on these alignments and quantify them using approaches similar to RNA-seq by expectation maximization (RSEM), which uses expectation maximization to accurately quantify expression using short-read data [50].

#### (iv) Modification detection

RNA transcripts are known to be host to a much larger variety of base modifications than genomic DNA. Except for A-to-I modifications which are read by the RT enzyme as G and therefore appear in cDNA, RNA modification cannot be detected by standard cDNA sequencing as performed by Illumina, PacBio and ONT [51].

Direct RNA sequencing, which is now possible on ONT sequencers, therefore holds great potential for modification discovery (figure 4). To realize this potential both computational and experimental workflows will need to be developed and improved. Although anecdotal evidence exists that modification information can be extracted from ONT base and raw data, no experimental and computational workflows exist yet to systematically establish and validate the detection of the large variety of modifications present in RNA [28]. Furthermore, improvements to experimental workflows will have to reduce the RNA input requirements which currently limit direct RNA sequencing to large samples or cell lines.

The detection of DNA modifications using raw PacBio data may serve as a cautionary tale here [52]. While the detection of methylated bases was shown to be possible using raw PacBio data, this approach never managed to compete with Illumina-based bisulfite sequencing for methylation detection. However, direct RNA sequencing has the potential to detect RNA modifications for which currently no other sequencing assay exist and might therefore fill a unique niche in the genomic toolset.

## 5. Conclusion

There is little doubt in our minds that full-length transcriptome sequencing using long-read technologies is the future of transcriptome annotation because it has too many inherent advantages over short-read approaches.

A single accurate long read covering a full-length transcript can—base-accurately—determine its TSS, all splice sites and polyA site, thereby immediately identifying the isoform the transcript represents. By contrast, regular short-read RNA-seq protocols rarely detect TSSs and polyA sites and usually only covers a subset of splice-sites with each individual read, leaving the researcher with a large computational problem when trying to identify isoforms which often has no unambiguous solution.

We are confident that in the next few years, by addressing the challenges we describe here, long-read sequencing will make high-quality transcriptome annotations readily achievable within a reasonable budget. This will be of particular interest to researchers working on organisms that have not attracted the attention of large consortia. Unicellular eukaryotes, in particular, could benefit hugely from this approach. Going forward, using 10X Genomics or Drop-seq approaches paired with long-read sequencing technology would allow for the amplification and sequencing of full-length cDNA from single-cell organisms to generate detailed isoform-level transcriptome annotations. Processing tens of thousands of cells this way could help generate an atlas of unicellular eukaryotes and would vastly expand our knowledge of the diversity of eukaryotic life.

## Data accessibility

This article has no additional data.
